# Neutralization of Interferon-gamma is efficacious in a mouse model of HLH secondary to chronic inflammation

**DOI:** 10.1186/1546-0096-13-S1-O29

**Published:** 2015-09-28

**Authors:** G Prencipe, I Caiello, C Bracaglia, C de Min, F De Benedetti

**Affiliations:** 1 Bambino Gesù Children's Hospital, Unit of Rheumatology, Rome, Italy; 2 NovImmune SA, Geneva, Switzerland, Italy

## Introduction

Macrophage activation syndrome is a term used to identify hemophagocytic lymphohistiocytosis secondary to rheumatic diseases (rheuHLH). It is a severe, potentially fatal condition that occurs in the context of rheumatic diseases, particularly systemic juvenile idiopathic arthritis. It is part of secondary HLH forms, that are clinically and biochemically similar to primary HLH (pHLH), with generalized hypercytokinemia as a major feature. While the triggering mechanism behind pHLH is the defect in cytotoxicity, caused by mutations in genes encoding proteins required for lymphocyte and natural killer cell activity, the rheuHLH pathogenesis is not clearly understood.

## Objectives

Based on data obtained in animal models of pHLH, showing that interferon-gamma (IFN-γ) neutralization reverts the disease, we aim to demonstrate, in a murine model of rheuHLH, the role of IFN-γ and the efficacy of an anti-IFN-γ antibody.

## Materials and methods

A mouse model of rheuHLH (Strippoli R, Arthritis Rheum 2012), recently developed in our laboratory, relying on an exaggerated response to toll-like receptor ligands in mice transgenic for the pro-inflammatory cytokine interleukin-6 (IL6TG), has been used to evaluate levels of IFN-γ and the therapeutic potential of a rat neutralizing IFN-γ antibody (XMG1.2, BioXcell, USA).

## Results

LPS-treated IL-6TG mice showed an exaggerated inflammatory response, with significantly higher IFN-γ mRNA expression levels in liver and spleen, compared to LPS-treated WT mice. Moreover, we observed a significant increase in the expression of genes known to be induced by IFN-γ, such as CXCL9, CXCL10 and H2-Aa (class II antigen A, alpha), in the spleen and in the liver from LPS-treated IL-6TG mice compared to WT mice. IFN-γ neutralization studies have revealed that, in LPS-injected IL-6TG mice, anti-IFN-γ treatment significantly improves survival and body weight recovery, compared to control antibody-treated animals. Furthermore, a significant reduction in ferritin levels was observed in mice treated with anti-IFN-γ Ab, compared to control antibody-treated animals. Finally, a significant reduction in the mRNA expression of IFN-γ-induced genes was observed in spleen and liver from mice treated with anti-IFN-γ Ab.

## Conclusion

These data demonstrate that the IFN-γ pathway is up-regulated in LPS-treated IL-6TG mice, compared to the wild type mice. Neutralization of IFN-γ significantly improves survival and clinical hematological parameters. These results provide insights into the pathophysiology of rheu-HLH, further support the hypothesis that IFN-γ is the common mediator of all HLH forms and provide the rational for the therapeutic use of a monoclonal anti- IFN-γ antibody in this fatal disorder.

